# Genetic Diversity of Enterovirus A71, India

**DOI:** 10.3201/eid2101.140743

**Published:** 2015-01

**Authors:** Vinay K. Saxena, Sudhir Sane, Sushma S. Nadkarni, Deepa K. Sharma, Jagadish M. Deshpande

**Affiliations:** Enterovirus Research Centre, Mumbai, India (V.K. Saxena, S.S. Nadkarni, D.K. Sharma, J.M. Deshpande);; Jupiter Hospital, Thane, India (S. Sane)

**Keywords:** Enterovirus A71, acute flaccid paralysis, hand, foot and mouth disease, phylogenetic analysis, viruses, India

## Abstract

We have identified circulation of 3 genogroups of enterovirus (EV) A71 in India. A new genogroup (proposed designation G) was discovered during this study. We isolated genogroups D and G in wide geographic areas but detected subgenogroup C1 only in 1 focus in western India. A systematic nationwide search for EV-A71 is warranted.

Enterovirus A71 (EV-A71; *enterovirus species A*, genus *Enterovirus*, family *Picornaviridae*) was first isolated in 1969 from the cerebrospinal fluid of a patient with encephalitis in California, USA. EV-A71 is known to cause encephalitis; meningitis; hand, foot and mouth disease (HFMD); and acute flaccid paralysis (AFP) (*1*,*2*). EV-A71 epidemic activity has increased substantially throughout the World Health Organization South-East Asia and Western Pacific Regions since the 1997 outbreak of HFMD with severe neurologic complications and high case-fatality rates reported in Sarawak and peninsular Malaysia (*3*). In many countries, EV-A71 circulated for several years before large-scale outbreaks were reported (*4*). In China, several hundred thousand cases of HFMD have been reported in recent years (*5*). In India, the epidemiology of EV-A71 has remained largely unexplored. A small outbreak of HFMD in Kerala in 2003 and 36 (42%) of 87 encephalitis cases reported in western Uttar Pradesh during July 2004–November 2006 were attributed to EV-A71 infections only on the basis of serologic evidence (*6*,*7*). Isolation of EV-A71 from a patient with AFP in India was reported for the first time in 2001 (*8*). Recently, 2 research groups have reported frequent isolation of EV-A71 from persons with AFP in Uttar Pradesh, Karnataka, and Kerala (*9*,*10*).

EV-A71 strains isolated worldwide are classified into 4 genogroups: A–D (*11*). Genogroups B and C have been differentiated into subgenogroups B0–B5 and C1–C5 (*11*). Bessaud et al. proposed 2 new genogroups in sub-Saharan Africa (genogroup E) and Madagascar (genogroup F) (*12*). Other new subgenogroups proposed recently include C4a, C4b, C6, C7, and B6 (*9*,*13*). We detected 14 EV-A71 among nonpolio enterovirus (NPEV) isolates from persons with AFP, HFMD, and encephalitis reported in Mumbai and surrounding areas during 2008–2012. The objective of this study was to evaluate the phylogenetic relationship of the Indian EV-A71 strains in the global context.

## The Study

We studied 561NPEV isolates obtained from 2,530 AFP patients, 89 from 383 HFMD patients, and 1 from 23 encephalitis patients in Mumbai and surrounding areas during 2008–2012. Partial sequencing viral protein 1 (VP1) was used to identify the NPEV (sero) types as described previously (*14*). Fourteen EV-A71 isolates were thus identified: 10 from AFP patients, 2 from HFMD patients, and 1 each from patients with encephalitis and febrile illness. Five EV-A71 isolates identified at various times since 2002 from northern Indian states were also included in this study (J. Deshpande, unpub. data). The VP1 region of EV-A71–positive isolates was sequenced as described previously (*15*). Sequencing was done by using Big Dye Terminator v3.1 Cycle Sequencing kit in accordance with the manufacturer’s instructions (Applied Biosystems, Foster City, CA, USA). Sequences were resolved on ABI 3130*xl* Genetic Analyzer (Applied Biosystems) and edited by using Sequencher v4.10.1 (Gene Codes, Ann Arbor, MI, USA). Complete VP1 sequences were aligned by using ClustalW (http://www.ebi.ac.uk). Phylogenetic analysis was conducted in MEGA5 by using maximum-likelihood and neighbor-joining methods (http://www.megasoftware.net). Genetic distances were calculated by Kimura 2-parameter method. A genotype was defined by >85% nt sequence similarity in the complete VP1 region (*12*). Sequences were deposited in GenBank under accession nos. KF906416–KF906434.

Median age of patients from whom the EV-A71 strains were isolated was 28 months; 10 (56%) were girls (Table 1). Three EV-A71–positive patients reported in Mumbai had lived in Uttar Pradesh. EV-A71–positive HFMD cases were non-neurologic, and the encephalitis patient recovered completely without neurologic deficit. Medical examination results 60 days after onset were not available for the AFP patients.

**Table 1:** Characteristics of enterovirus A71–positive patients, India*

Patient ID	Age, mo/sex	Clinical disease	Onset date	Sample type tested	Onset to sample, d	Patient location, city, state
V08–179	31/M	AFP	2008 Jan 14	Feces	32	Mumbai, MH
BMC08-C20	12/M	Fever	2008 Aug 2	Feces	2	Mumbai, MH
V08–5327	37/F	AFP	2008 Nov 7	Feces	2	Mumbai, MH†
V10–4243	66/F	AFP	2010 Aug 3	Feces	38	Kanpur, UP‡
V11–913	30/F	AFP	2011 Feb 26	Feces	6	Mumbai, MH
V11–2209	40/F	AFP	2011 Apr 25	Feces	10	Jaunpur, UP‡
V11–6064	26/F	AFP	2011 Aug 2	Feces	20	Mumbai, MH
V11–4998	73/F	AFP	2011 Aug 9	Feces	12	Mumbai, MH
V11–12176	50/F	AFP	2011 Oct 13	Feces	5	Mumbai, MH
V11–12755	12/F	AFP	2011 Oct 29	Feces	2	Thane, MH
V11-H31	9/M	HFMD	2011 Nov 7	Throat swab	2	Thane, MH
V12–104	34/F	AFP	2012 Mar 8	Feces	7	Mumbai, MH
V12-CN06	42/M	Encephalitis	2012 May 28	Cerebrospinal fluid	1	Thane, MH
V12-H05	12/M	HFMD	2012 Jul 21	Throat swab	2	Thane, MH
R-17928	9/M	AFP	2002 Jun 30	Feces	9	Jotiba Phule Nagar, UP
R-19153	10/M	AFP	2002 Aug 14	Feces	9	Rampur, UP
N03–522–2	NA	Healthy child	2003 Mar 26§	Feces	0	Bulandsahar, UP
V12–1719–2	14/F	AFP	2012 Jul 7	Feces	5	Delhi, DL
R-80135	12/M	AFP	2012 Sep 11	Feces	10	Darbhanga, BI

*AFP, acute flaccid paralysis; BI, Bihar; DL, Delhi; HFMD, hand, foot and mouth disease; MH, Maharashtra; UP, Uttar Pradesh. †Resident of Mumbai with a travel history to Azamgarh, UP. ‡Cases reported from hospitals in Mumbai. §Fecal sample collection date for healthy child.

**Figure 1:**
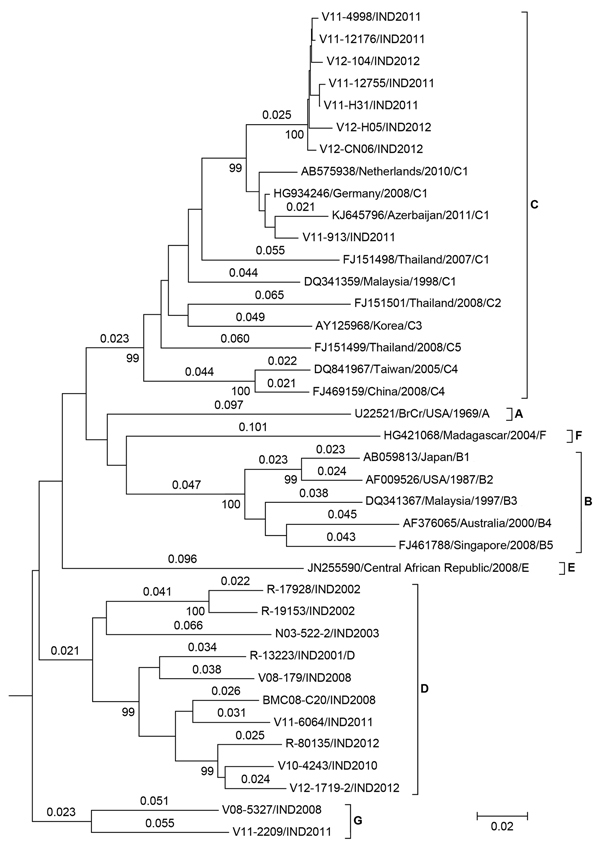
Neighbor-joining tree of enterovirus (EV) A71 using complete viral protein 1 (VP1) sequences. Genetic distances calculated by Kimura 2-parameter method are shown above the branches, and bootstrap values (1,000 replicates) are shown below. VP1 sequence of CA16 was used as the outgroup (not shown). Isolates V08–5327 and V11–2209 represent a new genogroup: G. Scale bar indicates nucleotide substitutions per site.

Nine isolates clustered with the strain R-13223-IND-01 (GenBank accession no. AY179600) (Figure 1), which was the only representative of EV-A71 genogroup D. A mean genetic distance of 11% and presence of multiple clusters within this genogroup indicated continuous evolution of genogroup D viruses over several years. Eight isolates (5 from AFP patients, 2 from HFMD patients, and 1 from an encephalitis patient) clustered with subgenogroup C1. Sequence similarity of >99% of 6 of the 8 isolates indicated a focal outbreak caused by C1 subgenogroup. Clustering of the subgenogroup C1 isolates with those from Germany, the Netherlands, and Azerbaijan suggested an epidemiologic link with the EV-A71 circulating in the European Region. V08–5327 and V11–2209 had sequence similarity of 89.5% between them and >18% divergence from all other known EV-A71 genogroups. We propose a new genogroup G designation for EV-A71 isolates V08–5327 and V11–2209. Table 2 shows estimates of evolutionary divergence of genogroup G in global context.

**Table 2:** Estimates of evolutionary divergence over sequence pairs between genogroups A–G of enterovirus A71, India*

Genogroup	Genogroup						
	A	B	C	D	E	F	G
A	X						
B	0.211	X					
C	0.195	0.205	X				
D	0.211	0.208	0.202	X			
E	0.232	0.216	0.196	0.187	X		
F	0.197	0.199	0.209	0.207	0.203	X	
G	**0.224**	**0.206**	**0.187**	**0.172**	**0.193**	**0.197**	**X**

*X indicates value not calculated because the diagonal values would be homologous comparisons (divergence 0%). Boldface indicates sequence divergence of genogroup G is >16% from all other genogroups.

**Figure 2:**
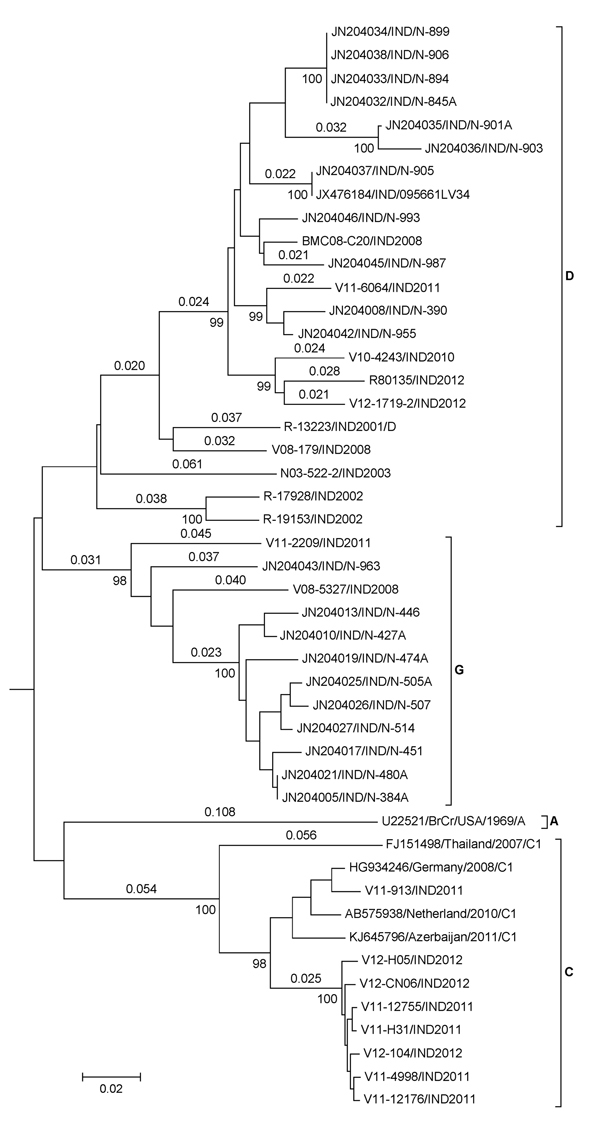
Neighbor-joining tree of enterovirus (EV) A71 strains isolated in India. Partial viral protein 1 (VP1) sequences (707 nt) were used for the analysis. Genetic distances calculated by Kimura 2-parameter method are shown above the branches, and bootstrap values (1,000 replicates) are shown below. India isolates clustered into 3 genogroups D and G and subgenogroup C1. VP1 sequence of CA16 was used as the outgroup (not shown). Scale bar indicates nucleotide substitutions per site.

To study the genetic diversity among EV-A71 strains isolated in India, we extracted partial VP1 nucleotide sequences of various lengths from GenBank. We detected 50 EV-A71 strains among NPEV isolates from AFP patients in Uttar Pradesh, Kerala, and Karnataka during 2007–2009 (*9*). Four EV-A71 isolates were reported from AFP patients from Uttar Pradesh studied during 2009–2010 (*10*). We used 21 sequences of isolates from Rao (*9*) and 1 of Laxmivandana (*10*) for determining the genetic relationship of EV-A71 isolated in India. Neighbor-joining tree using partial VP1 (707 nt) sequences (Figure 2) showed that 11 EV-A71 strains assigned to genogroup F by Rao et al. actually clustered within isolates of genogroup D, and the remaining 10 EV-A71 strains clustered within the new genogroup G. None of the EV-A71 isolates of Rao et al. (*9*) clustered in the subgenogroup C1 (Technical Appendix).

Bessaud et al. very recently reported (*12*) that 20 partial sequences reported by Rao et al. did not cluster into any known subgenogroup, which suggests more genogroups in India. Our current study confirms that some of them cluster within genogroup G. The remaining sequences from the study of Rao et al. need further analysis on the basis of longer nucleotide sequences.

## Conclusions

Our study reports the discovery of a new genogroup G of EV-A71 in India. Genogroups D and G may be endemic in India because they were isolated from AFP patients in wide geographic areas; however, these genogroups have not been implicated in any specific outbreaks. Moreover, the 2 genogroups appear to be indigenous to India because they have not been detected in any other country. EV-A71 strains of subgenogroup C1 were isolated only in 2011–2012, indicating very recent circulation. Subgenogroup C1 was associated with time- and space-clustered cases of AFP, HFMD, and encephalitis. Multiple genogroups and high sequence divergence within genogroups D and G showed that EV-A71 has been spreading across the country. Therefore, systematic efforts should be made to understand the impact of the virus on public health in India.

Technical Appendix Kimura 2-parameter genetic distances of enterovirus A71 isolates used in phylogenetic analysis.
